# Rapid Redox Hopping Charge Transfer and Electrochromism in a Multivariate Metal–Organic Framework

**DOI:** 10.1021/jacs.5c09275

**Published:** 2025-09-03

**Authors:** Benjamin Thomas, Sumanta Basak, Quinn Smith, Minliang Yan, Amanda J. Morris

**Affiliations:** † Department of Chemistry, 1757 Virginia Polytechnic Institute and State University, Blacksburg, Virginia 24061, United States; ‡ Macromolecules Innovation Institute, Virginia Polytechnic Institute and State University, Blacksburg, Virginia 24061, United States

## Abstract

Electrochromic materials exploit a change in molecular absorbance after an electrochemical redox event for applications, such as smart glass and segmented displays. Current applications use metal oxides; however, these materials are plagued by slow response times to potential changes. Herein, we investigate a metal–organic framework (MOF) film loaded with a molecular ruthenium redox carrier for its electrochromic capabilities. Upon the application of a potential jump, the native MOF was found to transport charge with an apparent diffusion coefficient, *D*_app_ of 8(±3) × 10^–9^ cm^2^/s. That said, only 55% of the redox centers, primarily those at the MOF surface, were converted. Using a multivariate (MTV) approach to incorporate sulfonate groups into the backbone of the MOF in addition to the redox carrier allowed for charge transport throughout the MOF with a *D*_app_ of around 1(±1) × 10^–7^ cm^2^/s and 100% conversion, the fastest reported diffusion coefficient in the redox hopping MOF literature to our knowledge. The sulfonated MOF exhibited a rapid electrochromic response, with coloration and bleaching times of approximately 1.29 and 1.33 s, respectively, at 400 mV overpotential. The sulfonate group is hypothesized to break ion pairs, allowing for higher ionic conductivity, which facilitates fast and complete charge transfer.

## Graphical abstract

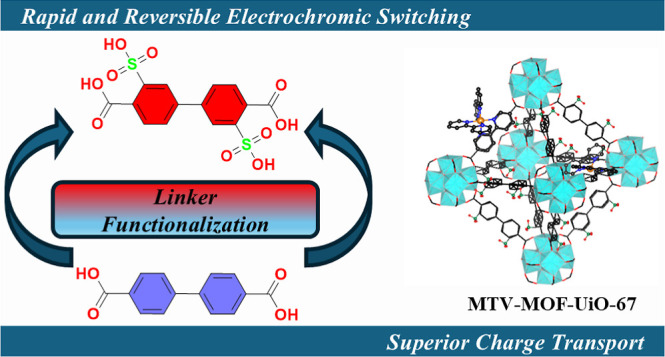

## 1 Introduction

Electrochromic materials are redox-active materials that undergo reversible optical changes when exposed to an applied potential bias. They are used in smart glass, segmented displays, and sensing materials.^1,2^ The most common electrochromics are metal oxides. During the reduction of these oxides, small ions intercalate into their crystal structure, stabilizing the injected electrons, which in some cases results in broad spectral, i.e., black absorbance, e.g., V_2_O_5_. However, the deintercalation process is slow; therefore, these materials are plagued by slow recovery of the initial spectral state. The metal oxides also lack versatility in tint color and synthetically tractable improvements in the rate of switching.^3^ Therefore, there is a desire to develop new electrochromic materials that exhibit similar stability, a more rapid color change response, and tunable colors beyond the broad spectrum.

Metal–organic frameworks (MOFs) are highly crystalline, porous frameworks consisting of metal oxide clusters bound by multidentate organic ligands.^4^ The versatility of metal ions and polydentate linkers that can be combined has led to MOFs being explored for a variety of applications, including gas storage,^5^ catalysis,^6–9^ drug delivery,^10^ and energy storage.^11^ MOF films grown on electrode surfaces were exploited for various electrochemical applications like electrocatalysis, batteries, and other energy storage applications.^12^ An understanding of the charge transfer mechanisms in these porous frameworks is critical to optimize structures to result in desired functions.^13^ Several mechanisms of charge transfer have been explored for porous frameworks, including through-space, through-bond, redox hopping, and guest-promoted transport.^14^ The MOF’s crystalline nature, coupled with the insulating properties of common MOF nodes, electronically isolates the redox units in locked positions in the framework. Thus, the dominant charge transfer mechanism in many MOF architectures is redox hopping, i.e., self-exchange electron transfer coupled to the diffusion of counterbalancing ions through the MOF pores.^15,16^ The diffusion of both the electrons and ions contributes to the overall charge transfer kinetics, giving an apparent diffusion coefficient, *D*_app_. The Morris group has previously investigated the charge transfer properties of MOFs through the Scholz Model.^16^ The Scholz model defines the movement of a charge through a microporous particle on an electrode surface. By using the Scholz model, the bottleneck of charge transport was determined to be the diffusion of the counterbalancing ions.^15^ On average, MOFs exhibit redox charge transfer with a *D*_app_ of 1–3 × 10^–10^ cm^2^/s.^17^ Previous reports of electrochromic MOFs incorporate redox active linkers, such as porphyrins or naphthalenediimide (NDI) derivatives. The incorporation of the electrochromic redox linkers allows absorbance to be used to monitor charge transfer within MOFs while determining the percent conversion of the redox centers. The Dincă’s research group presented a variety of NDI-MOFs in 2013 that showed multicolor electrochromic behavior that varied depending on the substituents linked to the linkers.^18^ The same research group has demonstrated that building MOFs using well-aligned electroactive linkers functionalized with phosphonate groups can establish effective pathways for both electron and proton transport within the framework.^19^ The Ott group synthesized three dipyrazole-terminated XDI (X = PM (pyromellitic), N (naphthalene), or P (perylene); DI = diimide) linkers with different diimide cores and incorporated them into Zn-XDI@FTO MOF thin films. Among them, Zn-PDI shows record-high coloration efficiency with excellent stability over 100 cycles due to electronically isolated PDI units enabling efficient Faradaic processes. Despite varying linker lengths, all three MOFs showed similar diffusion coefficients, indicating that cation-coupled electron hopping, enabled by their isoreticular structure and film orientation, dominates the transport mechanism.^20^

Herein, we report the redox-hopping-driven electrochromic response of UiO-67 MOF films with electrochromic Ru­(bpy)_2_(bpy-(COOH)_2_) (bpy = 2,2′-bipyridine; bpy-(COOH)_2_ = 5,5′-dicarboxylic acid-2,2′-bipyridine) (RuBPY) linkers incorporated along with the substituted biphenyl dicarboxylic acid in a multivariate (MTV) approach, as shown in Figure 1. MTV MOFs allow for the incorporation of multiple functional groups in unique spatial configurations that lead to improved activity greater than the sum of their parts.^21^ Sulfonic acid functionalization is a well-established strategy in polymer membranes and battery systems to enhance ion transport by disrupting ion pairs and enabling fast counterion migration. For example, sulfonated polymers like Nafion and sulfonated poly­(ether ether ketone) exhibit high proton conductivity due to −SO_3_H-mediated ion channels in fuel cell,^22,23^ while sulfonated binders and separators in batteries improve lithium-ion mobility and reduce ion pairing.^24^ Inspired by these systems, we applied sulfonation within a MOF (UiO-67) framework for the first time in the context of enhancing electrochromic performance. To our knowledge, this is the first report where sulfonation has been used to modulate redox hopping and accelerate apparent electron diffusion in a multivariate MOF. UiO-67 was selected as the MOF scaffold in this study not only due to its optical transparency, which avoids background interference in spectroelectrochemical measurements, but also because of its exceptional chemical and electrochemical stability under redox conditions. Its large pore size relative to that of UiO-66 enables more efficient ion diffusion, which is crucial for rapid charge balancing during electrochromic switching. Furthermore, the framework readily accommodates functionalized bipyridine linkers such as RuBPY and sulfonated derivatives through postsynthetic modification or direct synthesis, making it highly modular and tunable. These features make UiO-67 an ideal platform for investigating structure–function relationships in redox hopping and for benchmarking performance against previously reported redox-active MOFs. The Morris group has previously incorporated RuBPY, Ru­(bpy)_2_(bpy-(COOH)_2_) into UiO-67 to study photophysical properties.^15,25^ The incorporation of RuBPY is limited to one RuBPY per Zr node due to the confined pore space of the UiO-67 structure. The low ratio of redox centers can make the distance between them a barrier for charge transfer, making many of the RuBPY complexes electrochemically inaccessible. The native RuBPY-UiO-67 MOF films showed slow charge transfer rates with *D*_app_ of 8(±3) × 10^–9^ cm^2^/s while converting 55% of the film. Interestingly, adding sulfonate groups to the native BPDC linkers improved charge transport through the RUBPY-UiO-67-SO_3_H MOF, giving a *D*_app_ of 1(±1) × 10^–7^ cm^2^/s while converting 100% of the film. The rapid charge transfer and complete accessibility of redox centers enabled electrochromic applications where stable, reversible, and fast color switching was observed.

**Figure 1:**
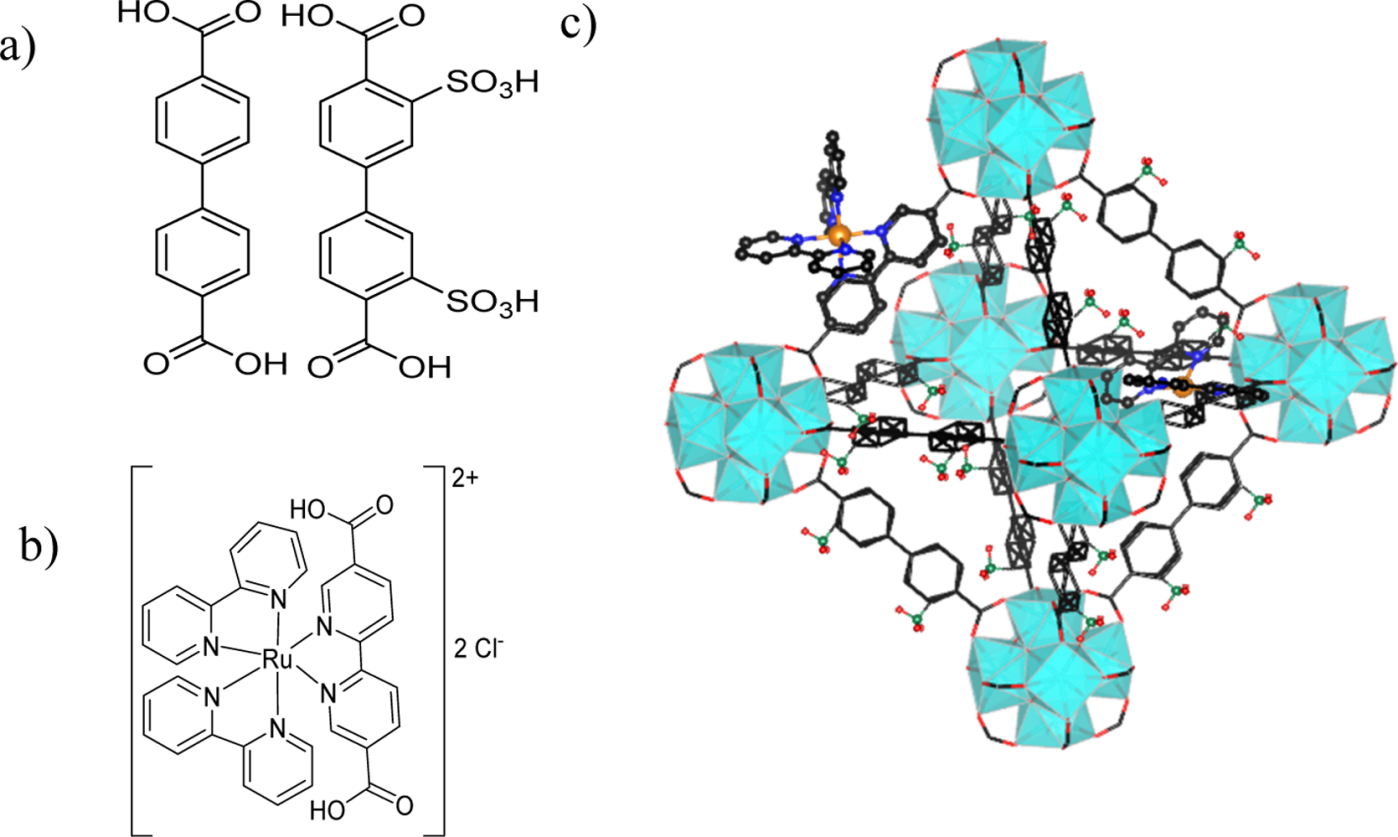
Linker structures of (a) BPDC and BPDC–SO_3_H, (b) RuBPY, and (c) single cage of RuBPY-UiO-67-SO_3_H.

## 2 Experimental Section

### 2.1 BPDC–SO_3_H Synthesis

Biphenyl-3,3′-disulfonyl-4,4′-dicarboxylic acid (BPDC–SO_3_H) was synthesized via a previously reported procedure (Figure 2).^26^ Briefly, 4,4′-dimethyl biphenyl was stirred and heated in concentrated sulfuric acid at 90 °C for 8 h. After the solution was cooled to room temperature, acetonitrile and dichloromethane were added to precipitate the intermediate product. The resultant precipitant, 3,3′-disulfonyl-4,4′-dimethylbiphenyl, was dried and dissolved in an aqueous sodium hydroxide solution before the addition of potassium permanganate. The purple solution was heated overnight at 90 °C, and the brown solid was filtered out. The filtrate was acidified with HCl and placed in the fridge for 10 h to precipitate the biphenyl-3,3′-disulfonyl-4,4′-dicarboxylic acid, which was then filtered and washed with 1 M HCl. ^1^H NMR: (400 MHz, DMSO-*d*_6_): δ 8.10 (s,2H), 7.85 (s,4H).

**Figure 2:**
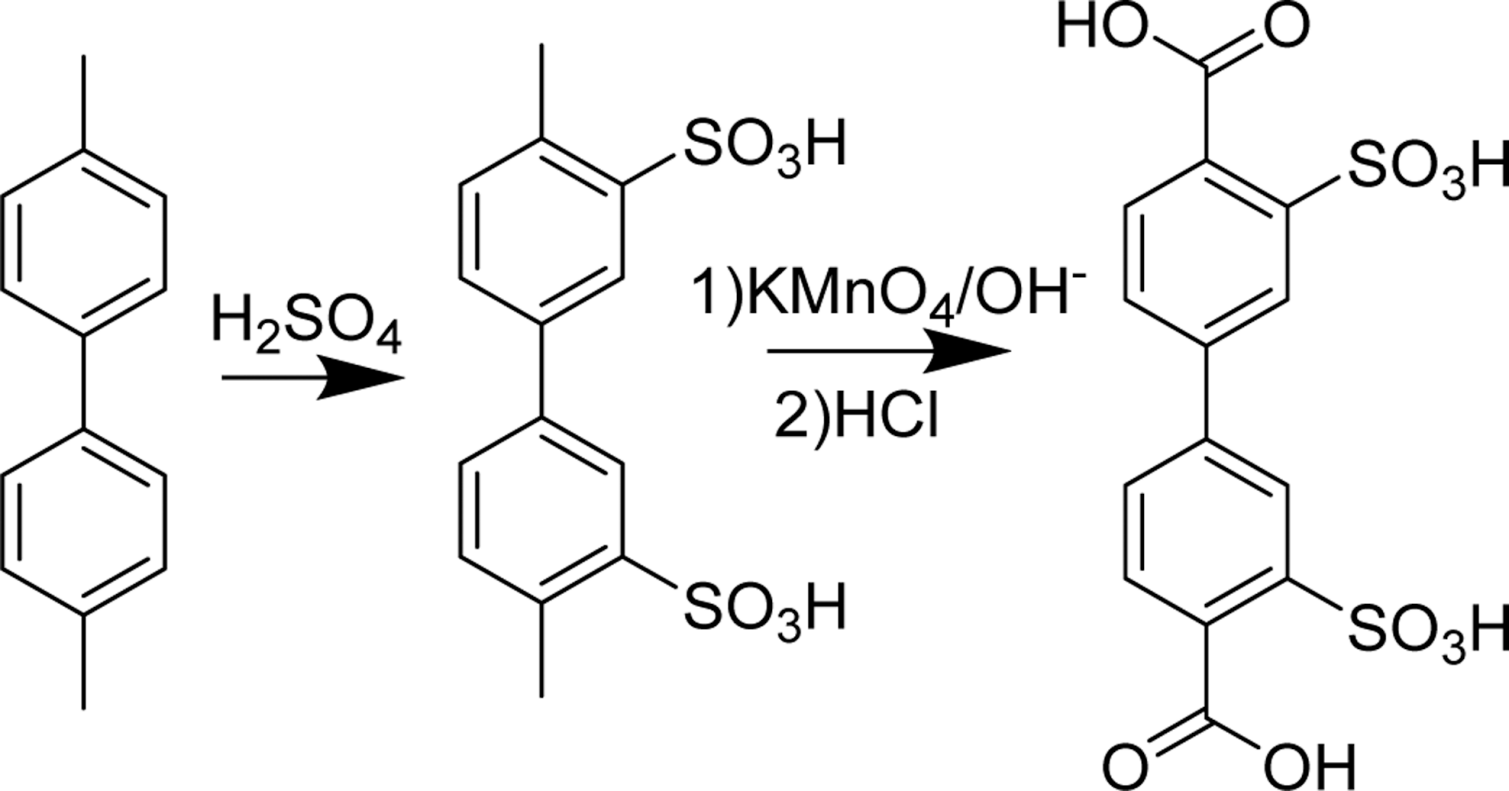
Synthesis procedure for BPDC–SO_3_H.

### 2.2 RuBPY Synthesis

RuBPY, (Ru­(bpy)_2_(bpy-(COOH)_2_) where bpy = 2,2′-bipyridine; bpy-(COOH)_2_ = 5,5′-dicarboxylic acid-2,2′-bipyridine, was synthesized via a previously reported procedure.^25^ Ru­(bpy)_2_Cl_2_ (200 mg, 0.30 mmol) and bpy-(COOH)_2_ (160 mg, 0.65 mmol) were added to a 3:1 ethanol/water mixture, and the mixture was refluxed overnight. The product was collected by rotary evaporation and recrystallized with methanol and diethyl ether. ^1^H NMR: (400 MHz, DMSO-*d*_6_): δ 9.00 (d, 2H), 8.88 (d, 2H), 8.84 (d, 2H), 8.53 (d, 2H), 8.23 (t, 2H), 8.17 (t, 2H), 8.01 (d, 2H), 7.86 (d, 2H), 7.79 (d, 2H), 7.60 (t, 2H), 7.51 (t, 2H).

### 2.3 RuBPY-UiO-67 Film Synthesis

RuBPY-UiO-67 films were prepared using a reported procedure.^27^ ZrCl_4_ (23 mg, 0.09 mmol), 4,4′-biphenyl dicarboxylic acid (21 mg), and RuBPY (20 mg, 0.08 mmol) were added to a 6-dram scintillation vial. Ten mL of DMF and 90 μL of difluoroacetic acid were added, and the vial was sonicated to dissolve the reagents. Fluorine-doped tin oxide (FTO) glass slides were soaked in piranha solution, followed by sonication in alconox, water and finally methanol before being placed in a 120 °C oven to dry. A cleaned FTO slide was placed in the vial, conductive side down. The vial was sealed and placed in a 120 °C oven for 24 h. The vials were removed and cooled to room temperature. The films were removed from the vial and rinsed with DMF and MeCN. The powder on the nonconductive sides was removed using a sodium hydroxide solution. The films were stored in MeCN for further use. RuBPY-UiO-67-SO_3_H films were prepared using the same procedure as above; however, the BPDC was substituted for BPDC–SO_3_H (40 mg).

### 2.4 Powder X-ray Diffraction (PXRD)

PXRD patterns for the MOF films were collected on a Bruker D8 Discover Wide Angle X-ray Diffractometer. The measurements were collected from 2θ values of 2° to 30° with a resolution of 0.02° at a rate of 0.25° per minute.

### 2.5 Electrochemical Measurements

Electrochemical measurements were conducted with a Pine Instruments WaveNow potentiostat using a three-electrode setup with the MOF film coated on an FTO slide as the working electrode, a Ag/AgCl reference electrode, and a platinum mesh counter electrode. All electrochemical experiments were done in 0.1 M LiClO_4_ in MeCN. For cyclic voltammetry measurements, the potential was swept from the open circuit potential (OCP) to 2000 mV vs NHE, which was well past the Ru^II/III^ couple. At least five sweeps were performed until the current response was stable, and the final cycle was reported. The scan rate was varied from 10 to 1000 mV/s to obtain various data points within this range for diffusion coefficient calculations.

### 2.6 Absorbance Measurements

Absorbance measurements were taken on a Cary UV–vis–NIR spectrophotometer from Agilent Technologies. Spectral scan measurements were taken in MeCN from 800 to 200 nm at a scan rate of 600 nm per minute. Spectroelectrochemical measurements were collected in a specialized quartz cuvette from Pine Research Instrumentation. A solution of 7.67 × 10^–5^ M RuBPY in MeCN was used to fill the cell. A honeycomb electrode from Pine Research Instrumentation with a platinum working electrode and counter electrode was placed in the cell along with a Ag/AgCl (saturated KCl) reference electrode. The cuvette was loaded into UV–vis and positioned so the light beam would go through the honeycomb electrode. An absorbance spectrum was taken to obtain the standard spectra of the RuBPY molecule. A potential of 1.5 V vs Ag/AgCl was applied for 5 min to oxidize the RuBPY at the electrode surface. The absorbance spectrum of the cell was then taken while holding the potential at 1.5 V vs Ag/AgCl until the spectrum stabilized, indicating complete oxidation.

For kinetic measurements, the absorbance at the selected wavelength was tracked, with data points collected every 0.033 s. The MOF film was placed in a glass cuvette, so the beamline would travel directly through the film. The cuvette was filled with 0.1 M LiClO_4_ in MeCN, and a Ag/AgCl reference electrode and platinum wire counter electrode were placed in the cell without blocking the beamline. A standard spectral scan was taken to determine the λ_max_, and a cyclic voltammogram at 50 mV/s was taken to determine the *E*_1/2_. A cyclic step chronoamperometry experiment was performed by applying 1800 mV vs NHE and subsequently applying the OCP; the two potentials were cycled up to 10 times as the absorbance at the λ_max_ was collected.

### 2.7 Raman Spectroscopy

Raman spectra were acquired using a 532 nm excitation laser with signal collection carried out by a CCD camera (Horiba XploRA PLUS). All measurements were performed under ambient conditions, utilizing a 10× objective lens for spectral acquisition.

## 3 Results and Discussion

### 3.1 Characterization of MOFs and UV–Vis Spectra

MOF films of RuBPY-UiO-67 and RuBPY-UiO-67-SO_3_H were solvothermally grown on transparent FTO electrode glass slides. The powder X-ray diffraction (PXRD) patterns for the synthesized MOFs closely match the simulated UiO-67 patterns, as shown in Figure 3a. SEM images show characteristic octahedral crystals, consistent with the morphology of UiO-type MOFs, as shown in Figure 3b,c. The films show several intergrown particles, and cross-section SEM gave film thickness of 1–3 μm (Figure S2).

**Figure 3:**
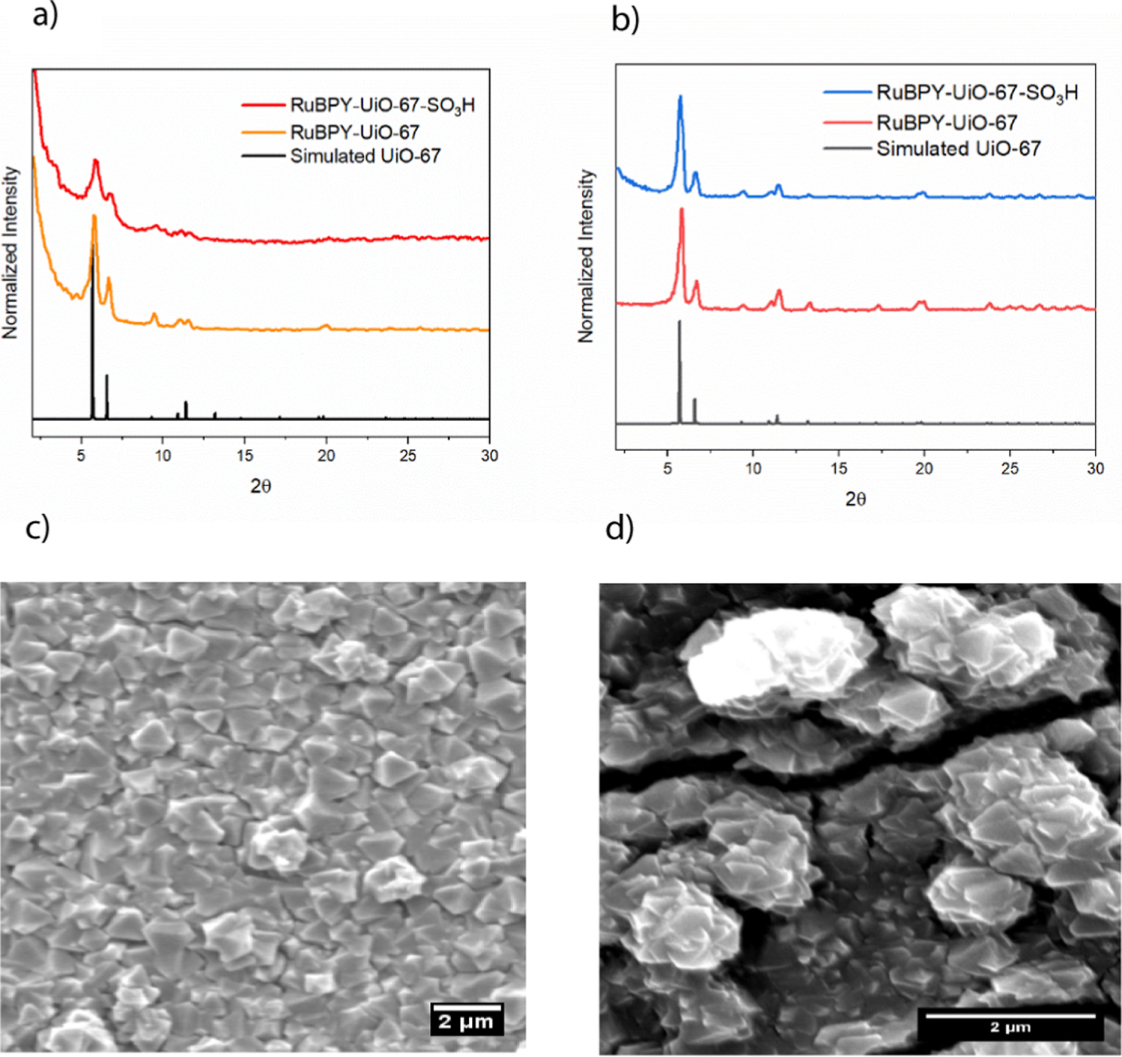
Characterization of MOF films: (a) PXRD patterns of RuBPY-UiO-67 and RuBPY-UiO-67-SO_3_H thin films as compared to a simulated pattern for UiO-67 (CCDC 1021002), (b) PXRD patterns of RuBPY-UiO-67 and RuBPY-UiO-67-SO_3_H powders as compared to a simulated pattern for UiO-67 (CCDC 1021002), (c) SEM image of a RuBPY-UiO-67 film, and (d) SEM image of a RuBPY-UiO-67-SO_3_H film.

Incorporating the RuBPY chromophore gives the UiO-67 MOF film an absorption profile similar to that of the molecule in homogeneous solution. RuBPY in the Ru^II^ oxidation state displays the classic metal–ligand charge transfer (MLCT) absorption band with a λ_max_ at 456 nm and a molecular extinction coefficient of 14670 M^–1^ cm^–1^. Upon oxidation, the electronic structure of the complex changes, weakening the metal-bpy interaction and resulting in a decrease in the MLCT band. The molar extinction coefficient of oxidized RuBPY in the Ru^III^ state at 456 nm is 978 M^–1^ cm^–1^ (Figure 4a,b).

**Figure 4:**
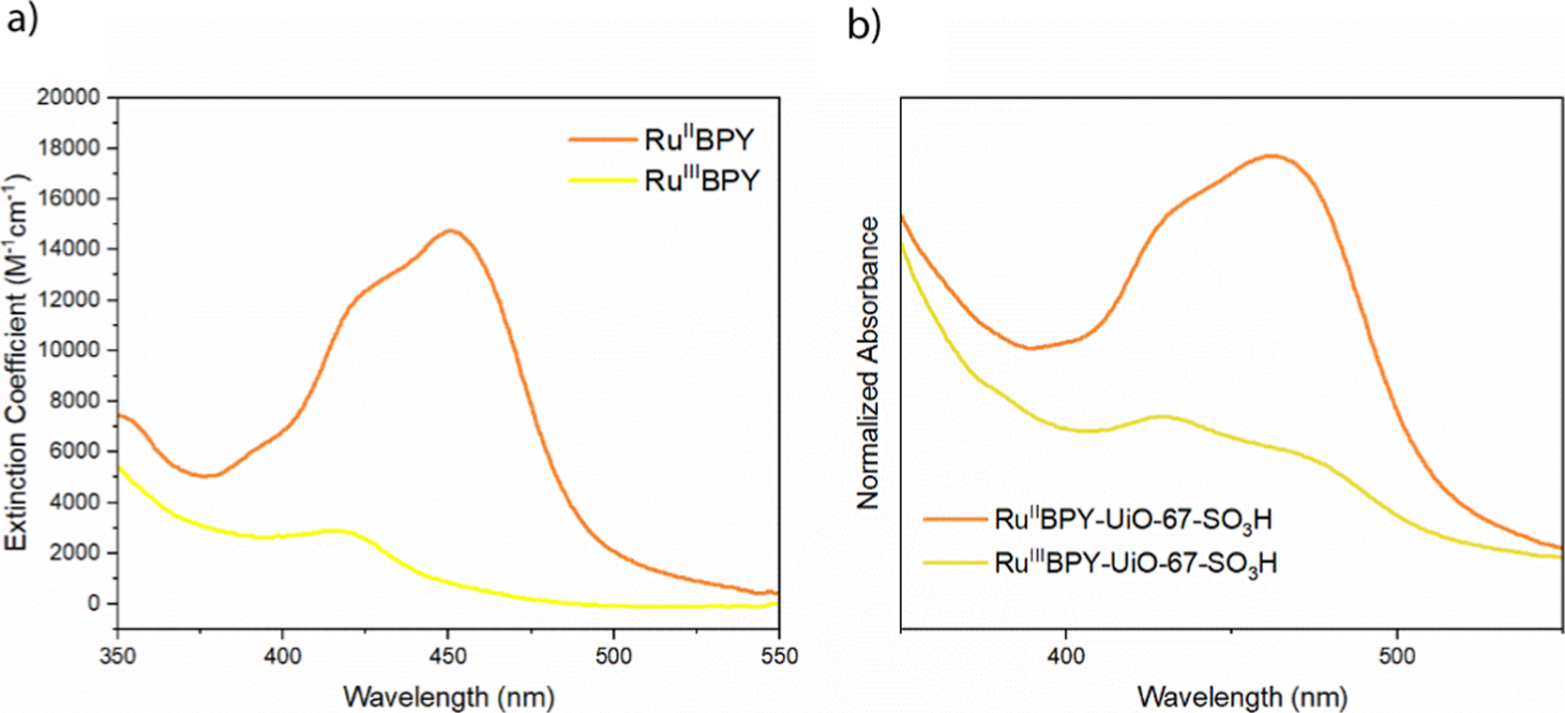
(a) Molecular absorbance spectra of the Ru^2+^(bpy)_2_ (bpy-COOH) (orange) and the oxidized counterpart, Ru^3+^(bpy)_2_(bpy-COOH) (yellow), and (b) the absorbance spectra of RuBPY-UiO-67-SO_3_H before (orange) and after (yellow) oxidation.

### 3.2 Electrochromic Response

To investigate the electrochromic response kinetics of the synthesized materials, we monitored the ratio of absorbance change as a function of time during repeated redox cycling (Figure 5). The switching behavior of both RuBPY-UiO-67 and its sulfonated analogue, RuBPY-UiO-67-SO_3_H, was examined under identical electrochemical conditions. In Figure 5, the absorbance at 456 nm is tracked for the MOF film when a potential of 1600 mV vs NHE was applied for 120 s followed by application of the original OCP of 50 mV vs NHE for 180 s.

**Figure 5:**
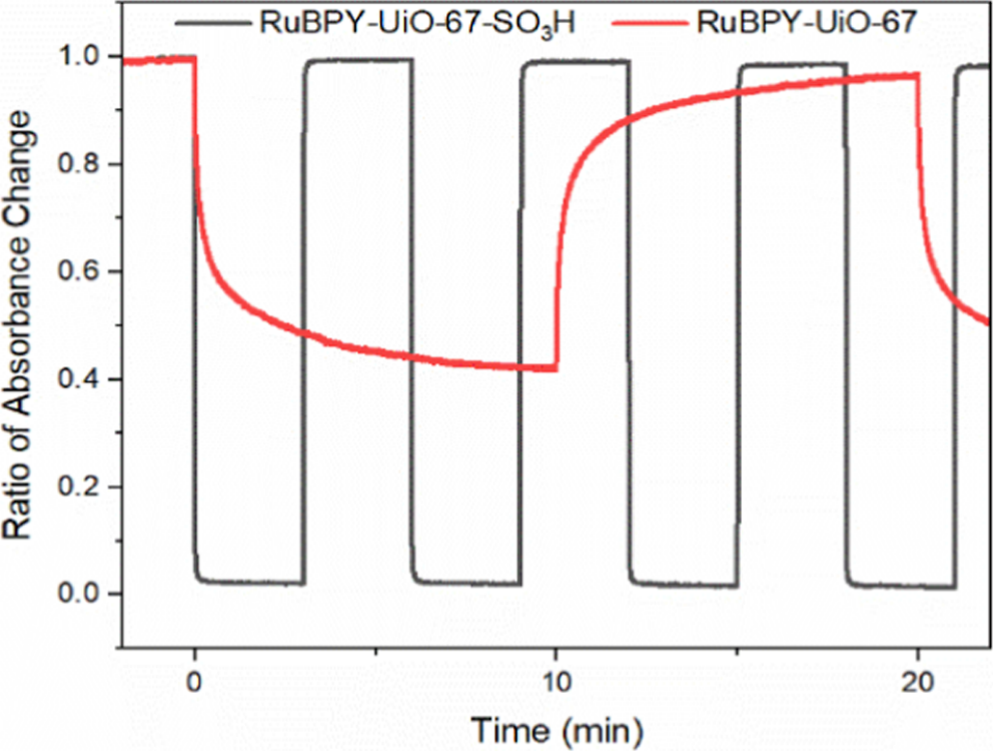
Change in absorbance as a function of time plots of RuBPY-UiO-67 (red) and RuBPY-UiO-67-SO_3_H (black) normalized to max absorbance in Ru^II^ state (1) and expected absorbance in Ru^III^ (0).

For RuBPY-UiO-67-SO_3_H, the forward (oxidation) and backward (reduction) processes exhibited nearly identical rates of 45 ± 2 min^–1^ and 46.3 ± 0.04 min^–1^, respectively, corresponding to time constants of approximately 1.33 and 1.29 s. The remarkable symmetry reflects highly reversible electrochromic behavior with minimal hysteresis between the oxidation and reduction steps (Video S1). The high-rate values also indicate fast electron transfer and ion mobility within the material, likely facilitated by the sulfonic acid functional groups, which enhance ionic conductivity and charge transport efficiency. Compared with traditional metal oxide electrochromic materials, our MOF demonstrates significantly faster switching speeds, even at lower overpotentials. For example, amorphous WO_3_·2H_2_O films typically exhibit slower coloration and bleaching times of 18.1 and 17.5 s, respectively, at an overpotential of approximately 550 mV.^28^ Similarly, sputtered WO_3_ films optimized for cycling durability show response and recovery times of 11 and 5 s at around 700 mV overpotential.^29^ Nickel–cobalt oxide thin films have achieved faster switching, with coloration and bleaching both occurring in under 4 s, around 300 mV overpotential.^30^ Vanadium-based systems such as VO_2_ and V_2_O_5_ composites may require up to 35 s to switch at 520 mV overpotential, while more advanced architectures like MoO_3_/V_2_O_5_ nanobilayers or Ti^4+^-doped V_2_O_5_ respond in 6–8 s at approximately 320 mV overpotential.^31,32^ Furthermore, when benchmarked against other MOF-based electrochromic systems, the performance of our MOF remains highly competitive (Tables S1, and S3).

In contrast, RuBPY-UiO-67 showed significantly slower switching dynamics, with forward and backward rates of 2.10 ± 0.07 min^–1^ and 2.16 ± 0.04 min^–1^, respectively. While the forward and backward rates remain relatively symmetric, the overall response time is more than an order of magnitude slower than that of the sulfonated analog. The results suggest that the absence of ion-conducting functionalities limits the electrochromic performance through restricted ion/electron mobility.

### 3.3 Measurement of Diffusion Coefficient (*D*_app_)

In the study of charge transfer in MOFs, the apparent diffusion coefficient (*D*_app_) is a key parameter used to characterize the overall charge transport. *D*_app_ accounts for two interconnected processes: electron hopping between redox centers (self-exchange) and counterion diffusion to balance the resulting charge. Various electrochemical techniques, including chronoamperometry, spectroelectrochemistry, and cyclic voltammetry, are commonly used to determine *D*_app_.^33^ These methods are favored due to the widespread availability of necessary equipment, such as a potentiostat and UV–visible spectrophotometer, and the relative ease of data interpretation. However, different techniques can yield varying *D*_app_ values due to differences in modeling assumptions and conditions. For instance, cyclic voltammetry assesses *D*_app_ based on small perturbations, reflecting initial thin-film conditions, whereas chronoamperometry and spectroelectrochemistry provide values that represent the overall thin-film behavior. Therefore, regardless of the method used, careful consideration is necessary to ensure accurate *D*_app_ measurements. In cyclic voltammetry, it is generally assumed that electron transfer kinetics are fast, ohmic drop is minimal, and peak currents reflect semi-infinite linear diffusion. However, high ohmic drop or capacitive currents can suppress peak currents, especially in resistive films or poorly conducting electrolytes. Spectroelectrochemistry assumes a uniform optical response throughout the film and that the electrochemical process is fully coupled to the optical signal. In chronoamperometry, the current decay is interpreted under the assumption of semi-infinite diffusion and negligible capacitive or ohmic contributions beyond early time points; thus, it is less sensitive to ohmic drop after the initial milliseconds. Chronocoulometry, particularly Anson plot analysis, assumes linearity of charge vs $\sqrt{t}$ and negligible background current; deviations may occur due to film heterogeneity or roughness, but the method remains robust for extracting apparent diffusion coefficients when carefully applied.^33^

#### 3.3.1 Spectroelectrochemistry

In this method, the change in extinction coefficients is used to track the conversion of the film from Ru^II^ to Ru^III^ by monitoring the absorbance as a function of time upon application of a potential bias. The diffusion of charge from the electrode surface throughout the MOF can be calculated using the rate of absorbance change according to a modified Cottrell eq (eq 1)­

1 $$\Delta A=\frac{2A_{\max}\sqrt{D_{app}t}}{d\sqrt{\pi }}$$

where Δ*A* represents the variation in absorbance, *A*_max_ denotes the maximum absorbance, *t* stands for time in seconds, and *d* indicates the thickness of the film. The sulfonated MOF film sample, shown in black (Figure 5), shows an immediate 100% conversion of Ru^II^ in the sample to Ru^III^. Using eq 2, the *D*_app_ for the RuBPY-UiO-67-SO_3_H was calculated to be 2.3(±3.7) × 10^–7^ cm^2^/s. The native sample showed a smaller change in overall absorbance at 456 nm over a 600 s period. Maximum conversion is reached when only 55% of ruthenium is oxidized to Ru^III^. The absorbance data suggest that not all the RuBPY centers are electrochemically accessible and converted from the ground state Ru^II^ to the oxidized Ru^III,^ and the charge is not fully diffusing throughout the framework.

To gain deeper insight into the ion transport dynamics governing the electrochromic switching of the synthesized MOFs, the diffusion coefficients for both the oxidation and reduction processes were extracted from the time-dependent absorbance profiles. These values offer a quantitative measure of the rate at which ions move through the material during redox reactions and are critical for evaluating the switching kinetics. We have found that the *D*_app_ for the forward step 2(±4) × 10^–7^ cm^2^/s is slower than the *D*_app_ for the backward step 2(±2) × 10^–6^ cm^2^/s. The difference in the rates for reduction and oxidation arises from ion transport mechanisms, charge interactions, and pore crowding.^34^ In oxidation, an additional negative ion must diffuse into the MOF to balance the positive charge, encountering increased crowding and ion-pairing interactions as the oxidation progresses inward. During the reduction process, the outermost Ru­(III) centers undergo reduction at first. As they are reduced, a negative ion diffuses from the MOF and moves into the bulk electrolyte solution. For the inner ruthenium centers, once reduced, the released ion follows a more accessible pathway to the bulk electrolyte solution. Furthermore, since all ruthenium centers along this path are already reduced and possess a lower effective charge, they exert less attraction, allowing ions to pass freely.

#### 3.3.2 Cyclic Voltammetry

Scan rate-dependent cyclic voltammetry (CV) can also be used to determine the *D*_app_ for MOF thin films on the surface of an electrode according to eq 2.^35^

2 $$\lambda_{e}=d_{f}\sqrt{\frac{Fv}{D_{app}RT}}$$

where λ_e_ is a dimensionless parameter and can be defined to relate the film thickness to the thickness of the electron diffusion layer, with *R* and *T* being the universal gas constant and absolute temperature, respectively. At low scan rates, *v*, the current response follows surface-based charge transfer, meaning the diffusion coefficient is fast enough to keep up with the rate of potential change meaning the peak current is linear with *v*. At higher scan rates, the current follows a diffusional charge transfer process as the *D*_app_ is too slow to keep up with the potential sweep meaning the current response is linear against *v* ^1/2^. The crossover scan rate can be used to determine the *D*_app_ of the sample using eq 3. At this point, λ_e_ is about 1, and a *D*_app_ can be found for a known film thickness calculated from cross-sectional SEM images.^35^ Figure 6 shows scan rate-dependent cyclic voltammetry of RuBPY-UiO-67 and RuBPY-UiO-67-SO_3_H.

**Figure 6:**
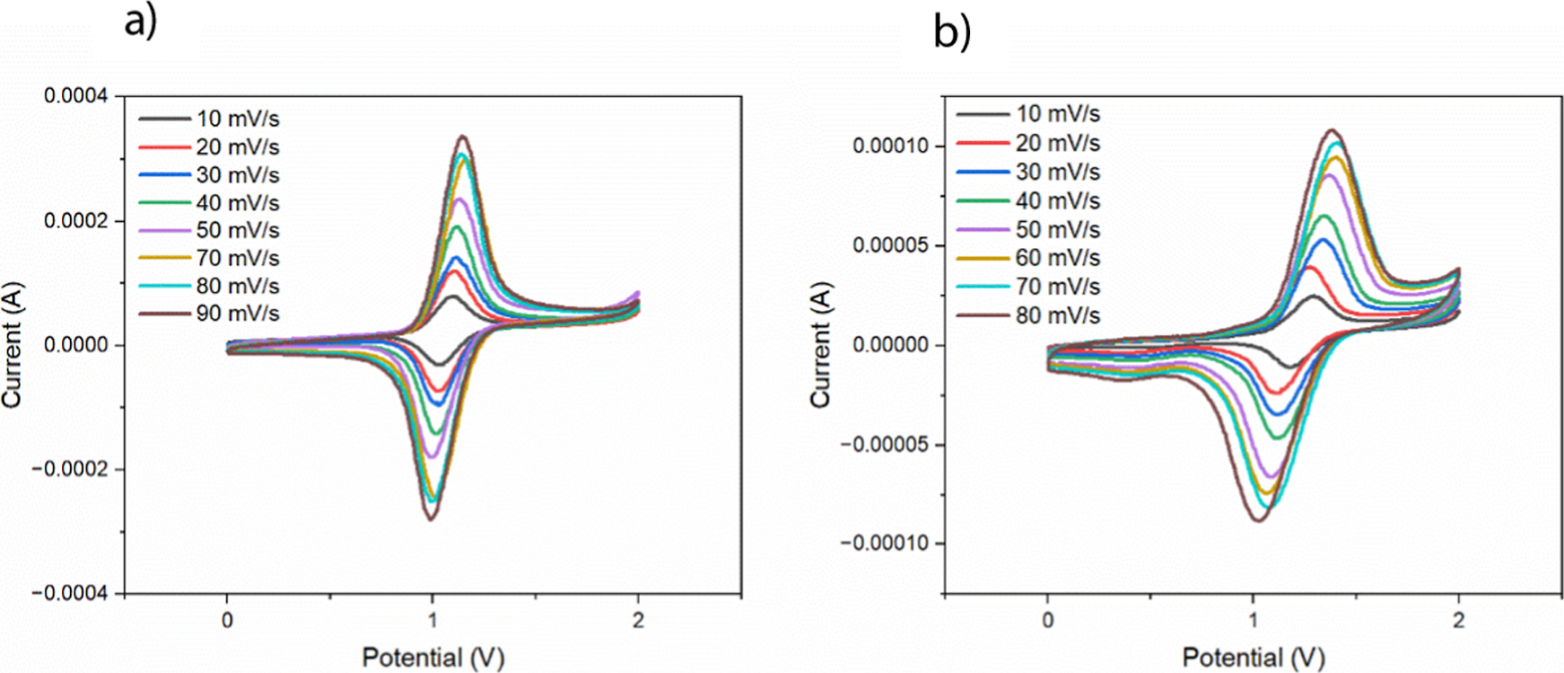
Cyclic voltammograms at varying scan rates for (a) RuBPY-UiO-67 and (b) RuBPY-UiO-67-SO_3_H.

The CVs of the native and the sulfonated MOF reveal distinct differences in their electrochemical behavior, particularly in terms of redox reversibility and electron transfer kinetics. The native MOF exhibits relatively small peak-to-peak separations across various scan rates, indicative of a more reversible redox process and faster electron transfer. In contrast, the sulfonated MOF displays significantly larger peak separations that increase with the scan rate, suggesting sluggish electron transfer kinetics and a quasi-reversible redox system. The *E*_1/2_ for both MOF films is shown to be 1.1 V vs NHE, which matches literature values for RuBPY.^27^ For the RuBPY-UiO-67-SO_3_H sample in 6b, we see a shift in the correlation with *v* to *v* ^1/2^ between the 40 and 50 mV/s scan rates in Figure 7a,b.

**Figure 7:**
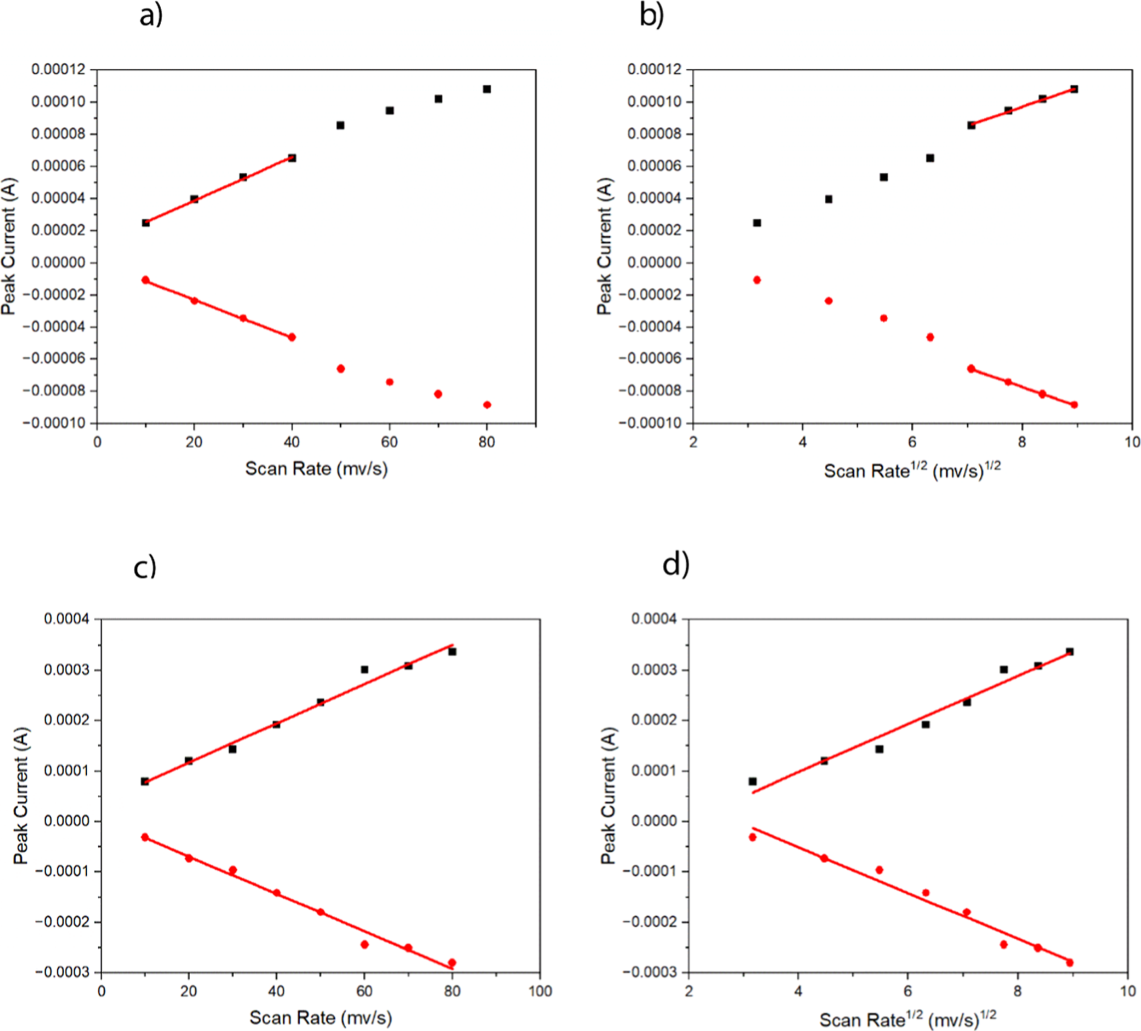
(a) Peak current vs scan rate for RuBPY-UiO-67-SO_3_H, (b) peak current vs square root scan rate for RuBPY-UiO-67-SO_3_H, (c) peak current vs scan rate for RuBPY-UiO-67, and (d) peak current vs square root scan rate for RuBPY-UiO-67.

Using a scan rate of 45 mV/s in eq 2 gives a *D*_app_ of 9(±4) × 10^–8^ cm^2^/s. For the native Ru-UiO-67, the scan rate dependence plot in Figure 6a never shows a transition to a diffusion-controlled process, suggesting one of two possibilities: the first is that the electron transport is so fast that the scan rate will only show a surface-bound process. However, increasing the scan rate up to 1000 mV/s, Figure S3, does not show a diffusion-based process, which would indicate a *D*_app_ that is extremely fast and unlike any similar literature values. The second explanation is that only the Ru centers close to the surface are being oxidized, and the rest of the material is electrochemically inactive.

#### 3.3.3 Potential Step Chronoamperometry

Chronoamperometry was also used to determine the *D*_app_. Diffusional electrochemical processes, namely, the time-dependent current after a potential jump, are accurately modeled using the Cottrell equation, eq 3.^33^

3 $$i=\frac{nFAC^{0}\sqrt{D_{app}}}{\sqrt{\pi t}}$$

where *i* is the current, *n* is the number of electrons transferred, *F* is Faraday’s constant (C/mol), *C* ^0^ is the concentration of redox species (mol/cm^3^), and *t* is the time (s). The plot of current versus 1/*t* ^1/2^ can be used to extract the *D*_app_. Chronoamperometry was performed on the MOF samples by applying 1800 mV vs Ag/AgCl to ensure complete oxidation of the redox active species. The current response for the films is shown in Figure 8a,b.

**Figure 8:**
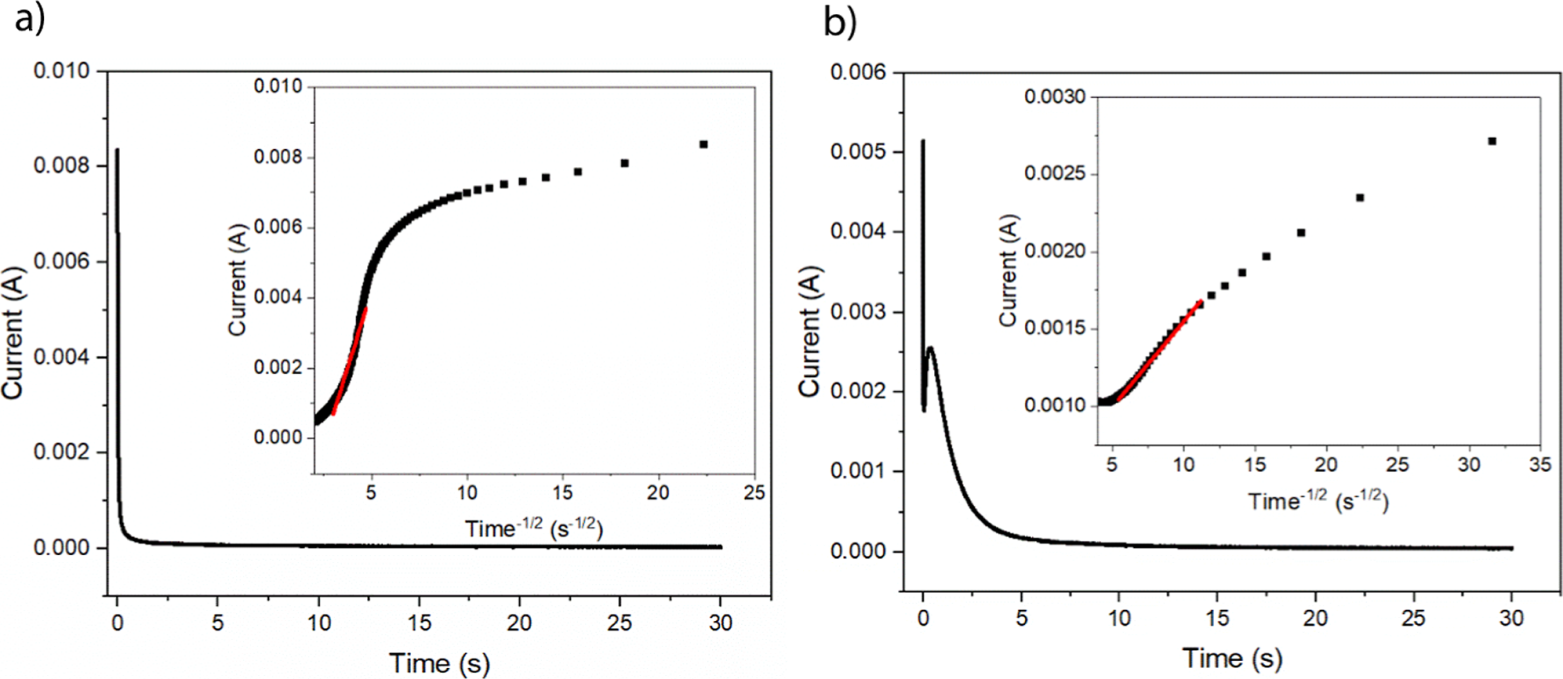
Chronoamperometry data for (a) RuBPY-UiO-67 and (b) RuBPY-UiO-67-SO_3_H. Insets: corresponding Cottrell plots for each species. The red lines indicate the linear region used to determine the slope.

Figure 8a presents the chronoamperometric response of RuBPY-UiO-67 over 30 s following a potential step, showing a sharp initial decrease in current consistent with diffusion-controlled kinetics. The inset displays the corresponding Cottrell plot, where the current is plotted against *t* ^–1/2^. A linear region (highlighted in red) confirms that the process adheres to the Cottrell behavior, indicating semi-infinite linear diffusion as the dominant transport mechanism. From the slope of this linear portion and applying the Cottrell equation, the *D*_app_ was calculated to be 8(±6) × 10^–9^ cm^2^/s. The response of RuBPY-UiO-67-SO_3_H shows a standard maximum in current at the initial potential application and then a subsequent rise in current after 0.6 s. RuBPY has only one accessible oxidation from Ru^II/III^ within the potential range applied, so a second electrochemical event is unlikely. A deviation from the Cottrell response suggests a drastic change in the electron transfer rate. The phenomenon was previously observed for redox active self-assembled monolayers of varying lengths bound to Ru redox centers.^36^ These fatty acid chains vary in length by as little as one carbon but show significant deviations in the electron transfer rates upon constant electrolysis. Finklea and Hanshew propose two different explanations for the nonlinear current response. The first is at high overpotentials, the resistance across the monolayer creates *iR* drop effects that lower the potential applied significantly enough to change the electron transfer rate. The second is that the varying distance of electron travel changes the diffusion rate. At short times, the redox centers at the electrode surface dominate the rate of electron transfer, while at longer times, the more distant redox centers control the current response. Thus, the MOF samples could have varying distances of redox centers leading to a variety of *D*_app_ values which would lead to the nonlinearity of the chronoamperometry plots. Additionally, in redox-conducting MOFs, charge transport is influenced by more than just diffusion. A recent study by Johnson et al. showed that both ion and electron migration driven by internal electric fields generated upon the application of a potential step play key roles in overall charge movement. Typically, the current decreases over time following a potential step, consistent with the diffusion-limited behavior described by the Cottrell equation. However, as the redox reaction proceeds, disparities between the rates of electron hopping and ion diffusion can cause an internal electric field to develop within the MOF film. This field enhances both electron and ion migration, resulting in a current that exceeds what diffusion alone would predict. The phenomenon may explain the sharp increase seen in the chronoamperogram. As the system moves toward equilibrium or as local ion concentrations are depleted, the internal electric field weakens, and the enhanced migration effect subsides, causing the current to return to a diffusion-controlled state.^37^ A Cottrell plot of the oxidation of RuBPY-UiO-67-SO_3_H is shown in Figure 8b. The *D*_app_ can be calculated using the slope of the line for the linear portion of the plot. The *D*_app_ calculated using this method was 8(±7) × 10^–8^ cm^2^/s, which is within error of the CV method determination.

The current plot can also be integrated to determine the charge transferred within the entire experiment. The charge can be plotted against *t* ^1/2^ which can be modeled to eq 4, also termed as the Anson equation, where *q* is the total charge passed. The Anson equation is commonly used for determining *D*_app_ as it is less influenced by non-Faradaic capacitive currents and the adsorption of electroactive species on the electrode surface. As the Anson method integrates current over time, it also naturally filters out noise.

4 $$q=\frac{2nFAc^{0}\sqrt{D_{app}t}}{\sqrt{\pi }}$$

From the slope of the Anson plot, Figure S4, the *D*_app_ was determined to be 1(±0.1) × 10^–7^ cm^2^/s.

#### 3.3.4 Discussion of the Observed *D*_app_s

Table 1 shows the calculated *D*_app_s using various techniques for RuBPY-UiO-67 and RuBPY-UiO-67-SO_3_H. The *D*_app_s are within error of each other, indicating the likelihood that the accurate *D*_app_ of the MOFs is close to the average of 8(±3) × 10^–9^ cm^2^/s for RuBPY-UiO-67 and 1(±1) × 10^–7^ cm^2^/s for RuBPY-UiO-67-SO_3_H. The data show that the RuBPY-UiO-67-SO_3_H has far higher conductivity than the RuBPY-UiO-67 counterpart. Remarkably, RuBPY-UiO-67-SO_3_H exhibits a diffusion coefficient on the order of 10^–7^ cm^2^/s which is two to 4 orders of magnitude higher than conventional MOFs and representing an exceptional enhancement in charge transport efficiency (see Table S2).

**Table 1:** Summary of Calculated *D*_app_s in cm^2^/s from Various Methods for MOF Thin Films

MOF thin film	CV	Cottrell (electrochemical)	Anson	Cottrell (spectroelectrochemical)	average
RuBPY-UiO-67		8(±6) × 10^–9^	10(±7) × 10^–9^	5(±1) × 10^–9^	8(±3) × 10^–9^
RuBPY-UiO-67-SO_3_H	9(±4) × 10^–8^	8(±7) × 10^–8^	1(±1) × 10^–7^	2(±4) × 10^–7^	1(±1) × 10^–7^

Given the ambipolar nature of redox hopping, i.e., both an electron and an ion need to diffuse, it is likely that the −SO_3_H groups modify electron or ion transport rates. Two theories were investigated to elucidate the role of sulfonate groups in charge transfer mechanisms. The first was that the BPDC–SO_3_H groups themselves were redox-active and could mediate charge transport. However, no electrochemical signature was observed for a –SO_3_H-derived UiO-67 within a reasonable electrochemical window compared to that of RuBPY as shown in Figure S5.

The second theory relates our structure to that of the polyelectrolytes. Polyelectrolytes are polymers consisting of repeating units that contain an ionizable species.^38^ When dissolved in solution, the ionic groups disassociate and create a charged polymer backbone. Polyelectrolytes with sulfate functional groups quickly deprotonate in solution because of their low p*K*_a_.^39^ Incorporating the negatively charged sulfate groups has improved the conductivity in batteries because the sulfate groups attract the Li^+^ in the LiClO_4_, freeing up the ClO_4_^–^ increasing the ionic conductivity.^40^ The native MOF lacking the charged functional groups on the biphenyl linkers would have a higher concentration of the contact ion pair in LiClO_4_ compared to RuBPY-UiO-67-SO_3_H.

To further support the Li^+^ interaction theory, Raman spectroscopy was employed to investigate potential structural and coordination changes in the sulfonated MOF framework before and after electrochemical experiments in a LiClO_4_-containing electrolyte (Figure 9). Raman spectroscopy was selected due to its sensitivity to vibrational modes associated with functional groups such as sulfonates, allowing us to monitor any changes in the local bonding environment of these groups resulting from electrochemical processes or ion coordination. In the sulfonated MOF, the stretching modes of the sulfonate group were clearly observed in the Raman spectrum at wavenumber values, e.g., ∼1027 cm^–1^ and ∼1120 cm^–1^.^41^ After electrochemical experiments, a noticeable red shift (∼34 cm^–1^) in these vibrational bands was detected. This red shift is attributed to coordination of Li^+^ ions from the supporting electrolyte with the oxygen atoms of the sulfonate groups. Such coordination weakens the S–O bond due to electron density redistribution, resulting in a lower vibrational frequency. The phenomenon is consistent with previous studies reporting Li^+^-induced shifts in sulfonate-containing materials.^42–44^

**Figure 9:**
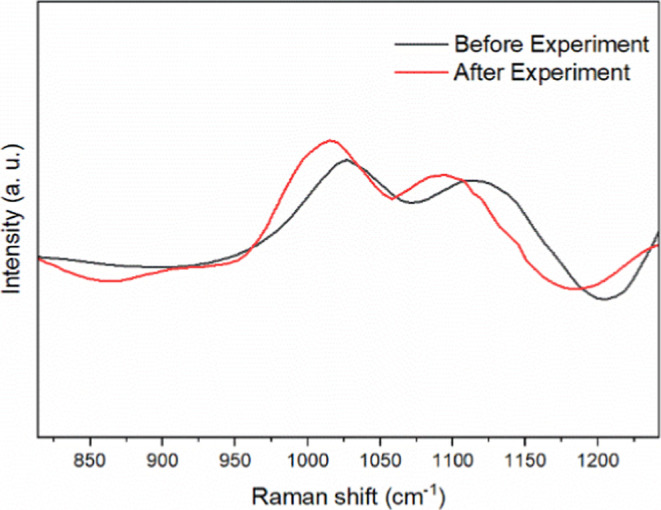
Raman spectra of RuBPY-UiO-67-SO_3_H MOF before (black) and after (red) electrochemistry experiments.

## 4 Conclusions

In this work, the electrochromic behavior of two MOFs, RuBPY-UiO-67 and RuBPY-UiO-67-SO_3_H, were investigated. The MOF thin films display noticeable alterations in their optical characteristics when their redox state changes. The RuBPY-UiO-67-SO_3_H demonstrates a fast coloration time of 1.29 s and a bleaching time of 1.33 s. Despite including the same redox active center, RuBPY, at the same concentration, the electrochromic response of RuBPY-UiO-67-SO_3_H was more complete (55% vs 100%) and occurred more rapidly. Cyclic voltammetry and spectroelectrochemistry support a lack of charge percolation through RuBPY-UiO-67 and surface-limited redox activity. Whereas the RuBPY-UiO-67-SO_3_H displayed classic diffusional transport with an apparent diffusion coefficient measured via a combination of methods to be 1(±1) × 10^–7^ cm^2^/s, to our knowledge, the largest coefficient reported for a MOF to date. We concluded that incorporating charged groups breaks ion pairs, enabling increased charge propagation throughout the framework as opposed to surface-limited oxidation. The results highlight the value of multivariate MOFs in electrochemical applications, where redox activity can be tuned significantly by easy modification of proximal but independent linkers.

## Supplementary Material





## Notes

NMR spectra, SEM image, electrochemical data (CV, Anson plot, chronoamperometry), and scan rate studies for RuBPY-UiO-67 derivatives and related materials (PDF)
Coloration and bleaching processes of RuBPY-UiO-67-SO_3_H@FTO upon electrochemical cycling in 0.1 M LiClO_4_ electrolyte solution (MOV)
