# Supplementary material for: Rapid Redox Hopping Charge Transfer and Electrochromism in a Multivariate Metal–Organic Framework
Source: J Am Chem Soc. 2025 Sep 3;147(37):33655–65. doi: 10.1021/jacs.5c09275 (PMC12447485; doi:10.1021/jacs.5c09275)
Supplement: Supplementary file 1 [file ja5c09275_si_001.pdf]

Supporting Information

Rapid Redox Hopping Charge Transfer and Electrochromism in a Multivariate Metal-Organic Framework

Benjamin Thomas [a], Sumanta Basak [a], Quinn Smith [a], Minliang Yan [b] and Amanda J. Morris [a b *]

[a]Department of Chemistry, Virginia Polytechnic Institute and State University, Blacksburg, Virginia 24061, United States

[b]Macromolecules Innovation Institute, Virginia Polytechnic Institute and State University, Blacksburg, Virginia 24061, United States

*Email: ajmorris@vt.edu

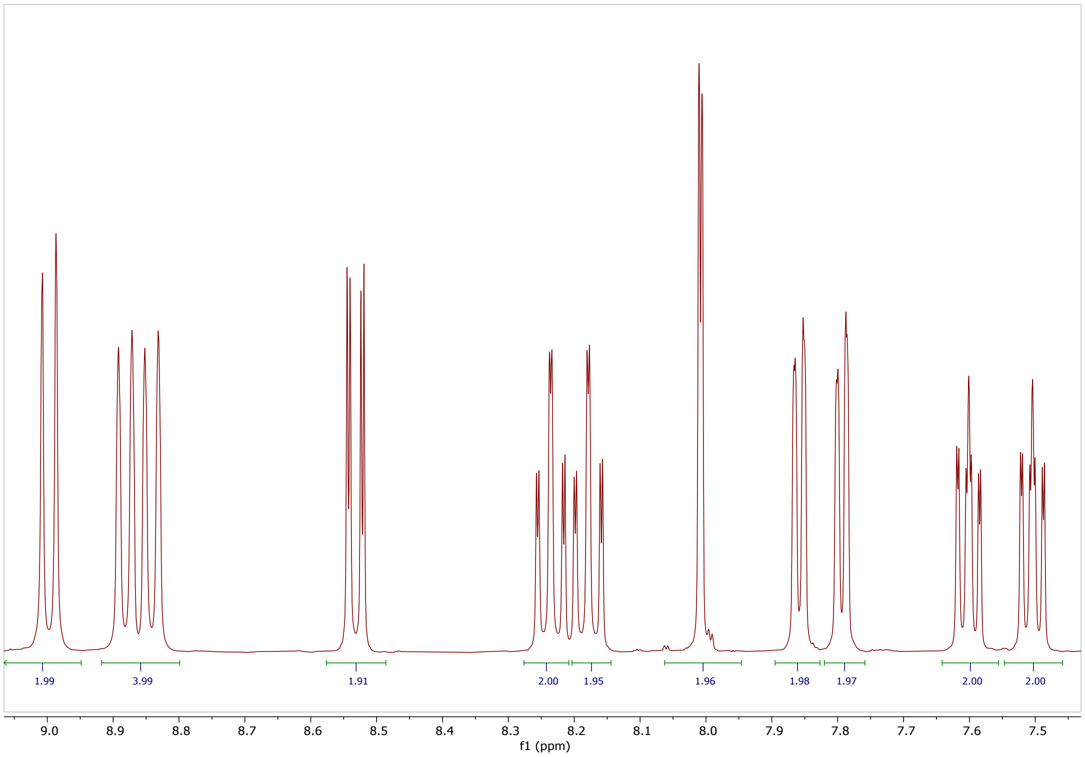

Figure S1: ^{1}H NMR spectrum of RuBPY in d_6-DMSO

(a) (b)

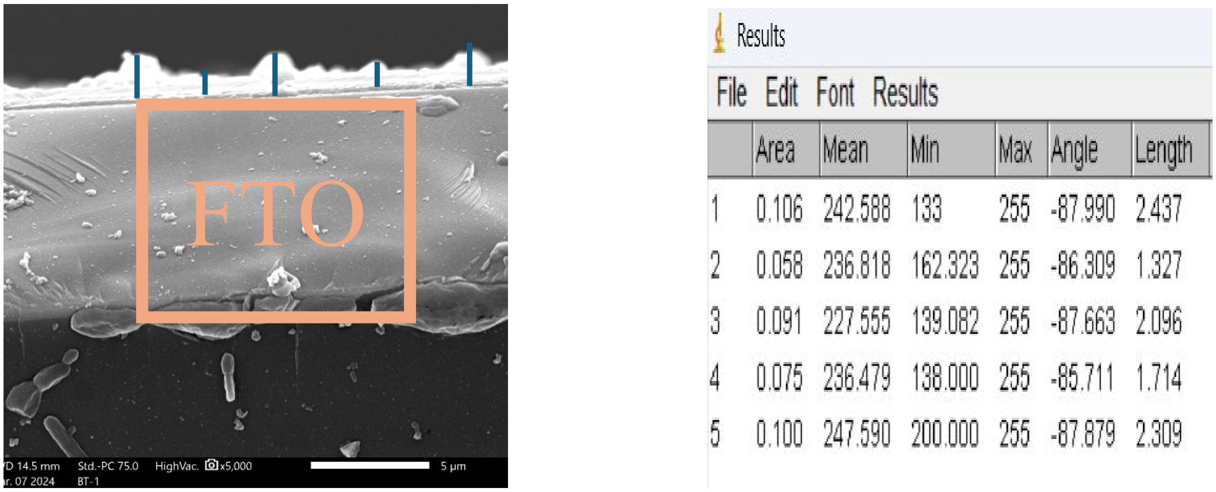

	Area	Mean	Min	Max	Angle	Length
1	0.106	242.588	133	255	-87.990	2.437
2	0.058	236.818	162.323	255	-86.309	1.327
3	0.091	227.555	139.082	255	-87.663	2.096
4	0.075	236.479	138.000	255	-85.711	1.714
5	0.100	247.590	200.000	255	-87.879	2.309

Figure S2: (a) Cross Section SEM of RuBPY-UiO-67-SO_3H film on FTO. The "FTO" label refers to the fluorine-doped tin oxide substrate, which serves as the conductive electrode; (b) ImageJ program was used to calculate the average thickness of the film.

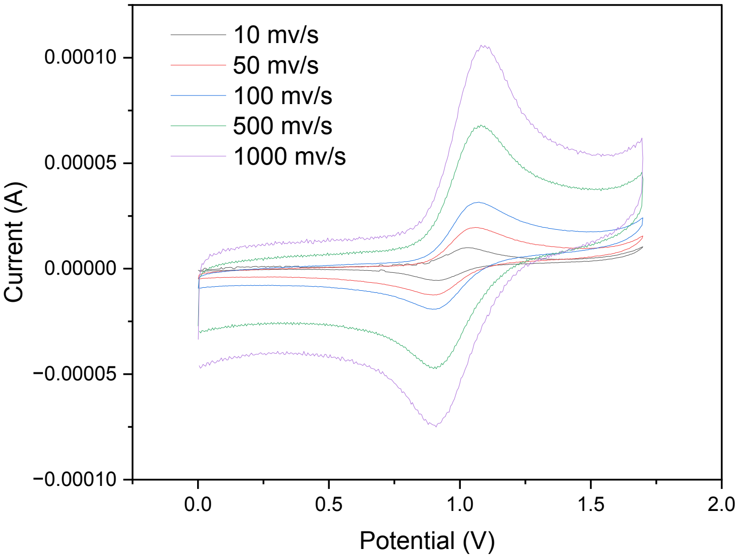

Figure S3: Scan Rate Dependence up to 1000 mV/s for RuBPY-UiO-67-SO3H. These measurements were carried out to evaluate the electron transfer kinetics and transport behavior within the MOF thin film. The persistence of well-defined redox peaks even at high scan rates indicates efficient charge transport and electrochemical reversibility.

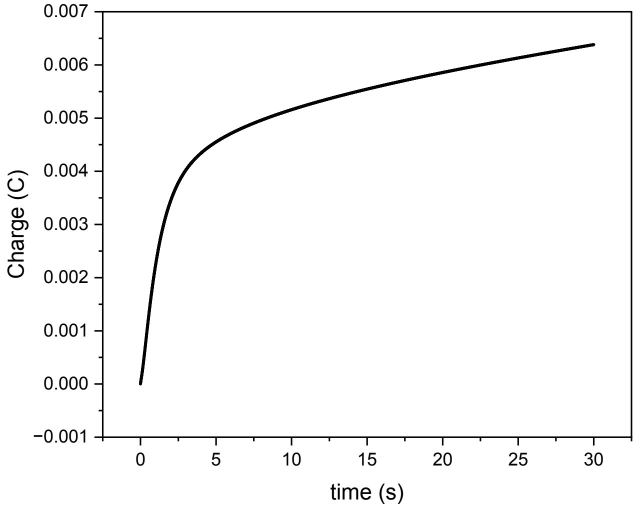

Figure S4: Anson Plot from current response of RuBPY-UiO-67-SO_3H where the slope of the linear region is used to extract apparent diffusion coefficient.

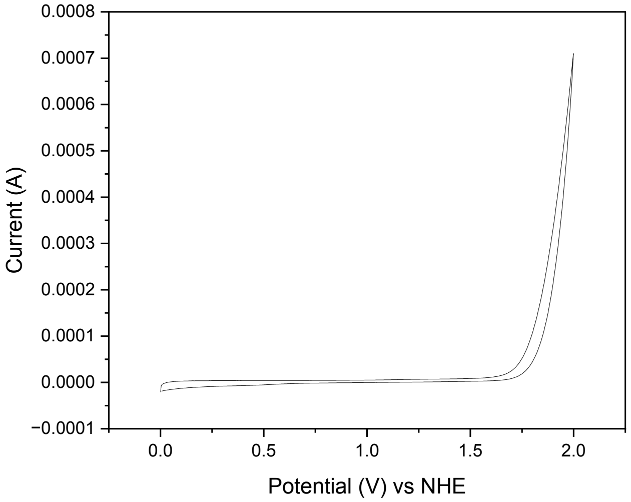

Figure S5: CV for catalyst free UiO-67-SO_3H. This control experiment demonstrates that the UiO-67-SO_3H framework alone does not exhibit redox activity under the studied potential window.

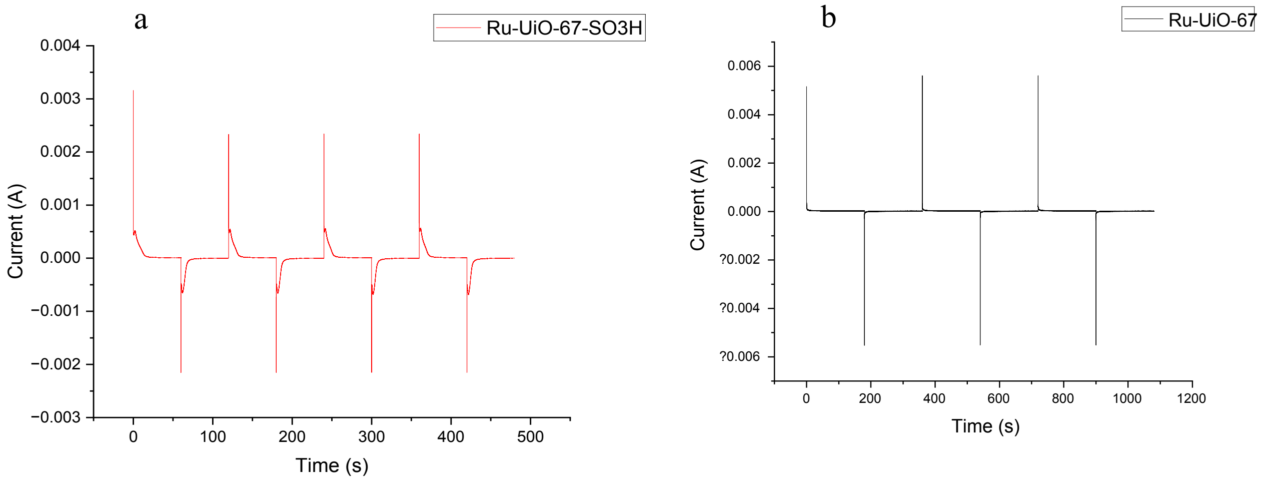

Figure S6: Cyclic step chronoamperometry for (a) RuBPY-UiO-67-SO_3H and (b) RuBPY-UiO-67 films on FTO. The sulfonated MOF (a) exhibits rapid and symmetric current transients, indicative of efficient electron hopping and ion transport. In contrast, the non-sulfonated analog (b) shows broader and delayed current responses, suggesting sluggish redox kinetics. These results highlight the functional advantage of sulfonation in enhancing electrochromic switching.

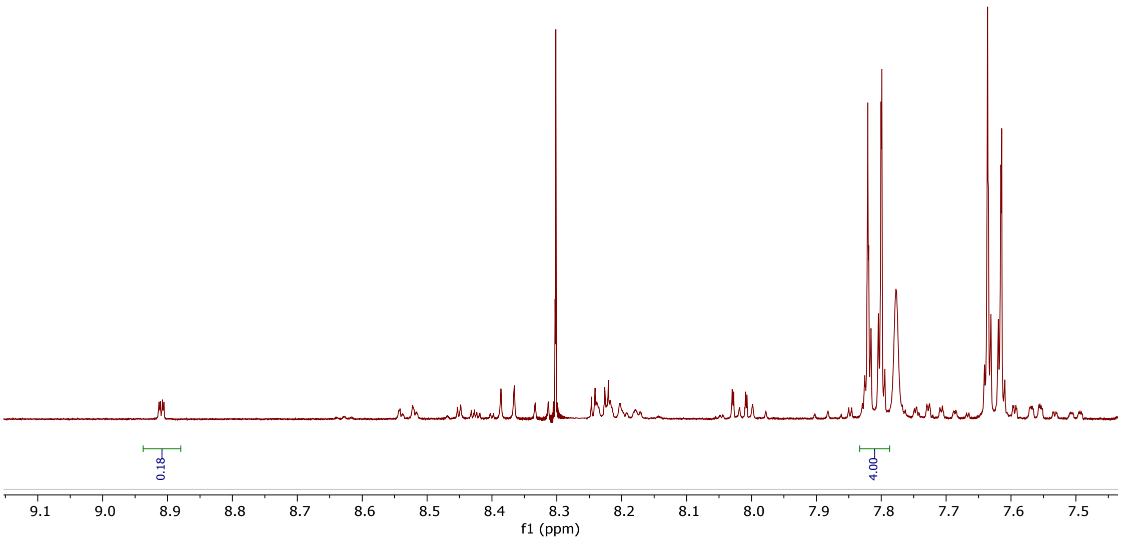

Figure S7: ^{1}H NMR spectrum of digested RuBPY-UiO-67-SO_3H

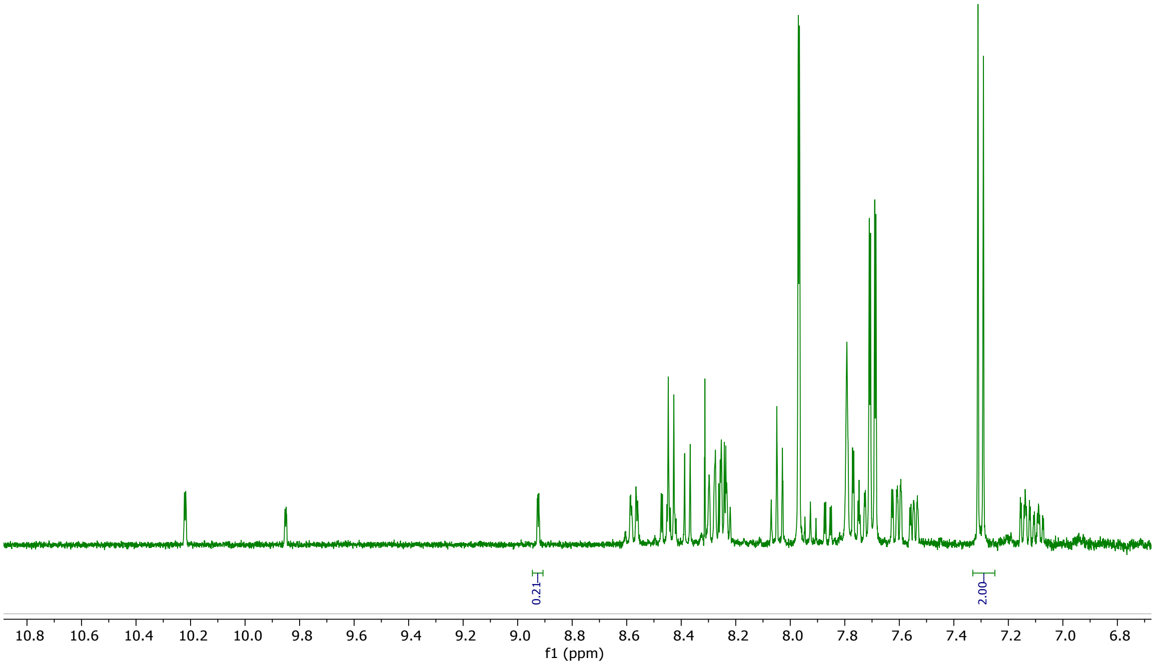

Figure S8: ^{1}H NMR spectrum of digested RuBPY-UiO-67

Table S1. Overview of reported MOF-based electrochromic materials in literature in comparison with the present study.

MOF Materials	Coloration Time (t_c) in second	Bleaching Time (t_b) in second	References
Zn (NDI-ATZ)	4.35	7.72	[1]
Zn-TCA (TCA= 4,4',4''-tricarboxytriphenylamine)	4.5	6.2	[2]
HKUST-1+ZnMOF-74	10	8	[3]
Cu-HHTP (HHTP = 2,3,6,7,10,11-Hexahydroxytriphenylene)	3.2	5.9	[4]
Ni- BINDI (BINDI = N,N-bis (5-isophthalic acid)-naphthalenediimide)	9.5	5.1	[5]
Ni-MOF-74	23	29	[6]
NU-901	5	12	[7]
RuBPY-UiO-67-SO_3H	1.29	1.33	This Work

Table S2. Overview of reported diffusion coefficients of MOFs in literature in comparison with the present study.

MOF System	Diffusion Coefficient (cm^2/s)	References
Metallocene-doped NU-1000	~10^{-10} – 10^{-9}	8
CoTCPP (TCPP = [5,10,15,20-(4-carboxyphenyl)porphyrin])	$7.55(\pm0.05)\times10^{-14}$	9
NU-1000	2×10^{-10}	10
Zr-(dcphOH-NDI)	$5.4(\pm1.1)x10^{-11}$	11
Ir-UiO-66	10^{-12}	12
Hemin-UiO-66	10^{-9}	13
NU-902	10^{-12}	14
RuBPY-UiO-67	$8(\pm3)x10^{-9}$	This Work
RuBPY-UiO-67-SO_3H	$1(\pm1)x10^{-7}$	This Work

Table S3. Overview of reported cycling number of MOFs in literature in comparison with the present study.

MOF	Electrolyte	Conductive substrate	Cycling number	References
Zn(NDI-H)	0.1 M $[(nBu)_4 N] PF_6$/DMF	FTO	25	[15]
Ni-NDISA	0.1 M $[(nBu)_4 N] PF_6$/DMF	FTO	10	[16]
Zn-DSNDI	0.1 M $[(nBu)_4 N] PF_6$/DMF	ZnO/FTO	10	[17]
Zr-BINDI	0.1 M $[(nBu)_4 N] PF_6$/DMF	ITO	100	[18]
Zn(NDIATZ)(NBU-3)	0.1 M $[(nBu)_4 N] PF_6$	FTO	6	[19]
NU-901 (Zr-TBAPy)	0.1 M $TBAPF_6$/ DCM	FTO	60	[20]
Mg-MOF-74	0.2 M $LiClO_4$/ MeCN	FTO	100	[21]
Zn-PDI	0.5 M KPF_6/ DMF	FTO	150	[22]
Ni-HITP205	0.1 M $LiClO_4$/PC (PC = Propylene carbonate)	FTO	100	[23]
Cu-TCA	0.1 M $LiClO_4$/PC	ITO	500	[24]
RuBPY-UiO-67-SO_3H	0.1 M $LiClO_4/CH_3CN$	FTO	100	This Work

References

(1) More, P. P.; Rathod, P. V.; Puguan, J. M. C.; Kim, H. All-in-One Display Device with Multicolor States Derived from NBU-3 MOF/Monoalkylated Viologen Hybrid Ionogel Material. *Dyes Pigm.* **2021**, *195*, 109730. https://doi.org/10.1016/j.dyepig.2021.109730.

(2) Liu, J.; Ma, X. Y. D.; Wang, Z.; Xu, L.; Wang, F.; He, C.; Lu, X. Metal–Organic Framework-Based Flexible Devices with Simultaneous Electrochromic and Electrofluorochromic Functions. *ACS Appl. Electron. Mater.* **2021**, *3* (3), 1489–1495. https://doi.org/10.1021/acsaelm.1c00097.

(3) Mjejri, I.; Doherty, C. M.; Rubio-Martinez, M.; Drisko, G. L.; Rougier, A. Double-Sided Electrochromic Device Based on Metal–Organic Frameworks. *ACS Appl. Mater. Interfaces* **2017**, *9* (46), 39930–39934. https://doi.org/10.1021/acsami.7b13647.

(4) Li, R.; Li, S.; Zhang, Q.; Li, Y.; Wang, H. Layer-by-Layer Assembled Triphenylene-Based MOFs Films for Electrochromic Electrode. *Inorg. Chem. Commun.* **2021**, *123*, 108354. https://doi.org/10.1016/j.inoche.2020.108354.

(5) Li, R.; Li, K.; Wang, G.; Li, L.; Zhang, Q.; Yan, J.; Chen, Y.; Zhang, Q.; Hou, C.; Li, Y.; Wang, H. Ion-Transport Design for High-Performance Na^+ -Based Electrochromics. *ACS Nano* **2018**, *12* (4), 3759–3768. https://doi.org/10.1021/acsnano.8b00974.

(6) Zhang, N.; Jin, Y.; Zhang, Q.; Liu, J.; Zhang, Y.; Wang, H. Direct Fabrication of Electrochromic Ni-MOF 74 Film on ITO with High-Stable Performance. *Ionics* **2021**, *27* (8), 3655–3662. https://doi.org/10.1007/s11581-021-04112-y.

(7) Kung, C.-W.; Wang, T. C.; Mondloch, J. E.; Fairen-Jimenez, D.; Gardner, D. M.; Bury, W.; Klingsporn, J. M.; Barnes, J. C.; Van Duyne, R.; Stoddart, J. F.; Wasielewski, M. R.; Farha, O. K.; Hupp, J. T. Metal–Organic Framework Thin Films Composed of Free-Standing Acicular Nanorods Exhibiting Reversible Electrochromism. *Chem. Mater.* **2013**, *25* (24), 5012–5017. https://doi.org/10.1021/cm403726v.

(8) Celis-Salazar, P. J.; Cai, M.; Cucinell, C. A.; Ahrenholtz, S. R.; Epley, C. C.; Usov, P. M.; Morris, A. J. Independent Quantification of Electron and Ion Diffusion in Metallocene-Doped Metal–Organic Frameworks Thin Films. *J. Am. Chem. Soc.* **2019**, *141* (30), 11947–11953. https://doi.org/10.1021/jacs.9b03609.

(9) Ahrenholtz, S. R.; Epley, C. C.; Morris, A. J. Solvothermal Preparation of an Electrocatalytic Metalloporphyrin MOF Thin Film and Its Redox Hopping Charge-Transfer Mechanism. *J. Am. Chem. Soc.* **2014**, *136* (6), 2464–2472. https://doi.org/10.1021/ja410684q.

(10) Goswami, S.; Hod, I.; Duan, J. D.; Kung, C.-W.; Rimoldi, M.; Malliakas, C. D.; Palmer, R. H.; Farha, O. K.; Hupp, J. T. Anisotropic Redox Conductivity within a Metal–Organic Framework Material. *J. Am. Chem. Soc.* **2019**, *141* (44), 17696–17702. https://doi.org/10.1021/jacs.9b07658.

(11) Johnson, B. A.; Bhunia, A.; Fei, H.; Cohen, S. M.; Ott, S. Development of a UiO-Type Thin Film Electrocatalysis Platform with Redox-Active Linkers. *J. Am. Chem. Soc.* **2018**, *140* (8), 2985–2994. https://doi.org/10.1021/jacs.7b13077.

(12) Chuang, C.-H.; Li, J.-H.; Chen, Y.-C.; Wang, Y.-S.; Kung, C.-W. Redox-Hopping and electrochemical behaviors of Metal–Organic framework thin films fabricated by various approaches. *J. Phys. Chem. C* **2020**, *124* (38), 20854–20863. https://doi.org/10.1021/acs.jpcc.0c03873.

(13) Shimoni, R.; He, W.; Liberman, I.; Hod, I. Tuning of redox conductivity and electrocatalytic activity in Metal–Organic Framework films via control of defect site density. *J. Phys. Chem. C* **2019**, *123* (9), 5531–5539. https://doi.org/10.1021/acs.jpcc.8b12392.

(14) Maindan, K.; Li, X.; Yu, J.; Deria, P. Controlling Charge-Transport in Metal–Organic frameworks: contribution of topological and Spin-State variation on the Iron–Porphyrin centered redox hopping rate. *J. Phys. Chem. B* **2019**, *123* (41), 8814–8822. https://doi.org/10.1021/acs.jpcb.9b07506.

(15) Wade, C. R.; Li, M.; Dincă, M. Facile deposition of multicolored electrochromic Metal–Organic framework thin films. *Angew. Chem., Int. Ed.* **2013**, 52 (50), 13377–13381. https://doi.org/10.1002/anie.201306162.

(16) AlKaabi, K.; Wade, C. R.; Dincă, M. Transparent-to-Dark electrochromic behavior in Naphthalene-Diimide-Based mesoporous MOF-74 analogs. *Chem* **2016**, *1* (2), 264–272. https://doi.org/10.1016/j.chempr.2016.06.013.

(17) Wu, X.; Wang, K.; Lin, J.; Yan, D.; Guo, Z.; Zhan, H. A thin film of naphthalenediimide-based metal-organic framework with electrochromic properties. *J. Colloid Interface Sci.* **2021**, *594*, 73–79. https://doi.org/10.1016/j.jcis.2021.02.083.

(18) Radha, G.; Roy, S.; Chakraborty, C.; Aggarwal, H. Electrochromic and photochromic behaviour in a single metal–organic framework containing a redox-active linker. *Chem. Commun.* **2022**, *58* (25), 4024–4027. https://doi.org/10.1039/d2cc00288d.

(19) Zhang, N.; Jin, Y.; Zhang, Q.; Liu, J.; Zhang, Y.; Wang, H. Direct fabrication of electrochromic Ni-MOF 74 film on ITO with high-stable performance. *Ionics* **2021**, *27* (8), 3655–3662. https://doi.org/10.1007/s11581-021-04112-y.

(20) Kung, C.-W.; Wang, T. C.; Mondloch, J. E.; Fairen-Jimenez, D.; Gardner, D. M.; Bury, W.; Klingsporn, J. M.; Barnes, J. C.; Van Duyne, R.; Stoddart, J. F.; Wasielewski, M. R.; Farha, O. K.; Hupp, J. T. Metal–Organic framework thin films composed of Free-Standing acicular nanorods exhibiting reversible electrochromism. *Chem. Mat.* **2013**, *25* (24), 5012–5017. https://doi.org/10.1021/cm403726v.

(21) Shiozawa, H.; Melnikova, Z.; Bastl, Z.; Peterlik, H.; Kalbac, M.; Frank, O. Electrochromic 2,5-Dihydroxyterephthalic acid linker in Metal−Organic frameworks. *Adv. Photonics* **2022**, *3* (4). https://doi.org/10.1002/adpr.202100219.

(22) Li, R.; Li, K.; Wang, G.; Li, L.; Zhang, Q.; Yan, J.; Chen, Y.; Zhang, Q.; Hou, C.; Li, Y.; Wang, H. Ion-Transport design for High-Performance NA+-Based electrochromics. *ACS Nano* **2018**, *12* (4), 3759–3768. https://doi.org/10.1021/acsnano.8b00974.

(23) Pan, L.; Li, R.; Zhang, C.; Lu, Z.; Li, K.; Zhang, Q.; Hou, C.; Li, Y.; Wang, H. Redox-Active NI(II) nodes induced electrochromism in a Two-Dimensional conductive Metal–Organic framework. *ACS Appl. Electron. Mater.* **2022**, *4* (6), 2915–2922. https://doi.org/10.1021/acsaelm.2c00388.

(24) Feng, S.; Wang, J.; Tong, Z.; Qu, H.-Y. Metal-organic framework thin films with diverse redox-active/inactive components for enhanced optical modulation and coloration efficiency. *Chem. Eng. J.* **2022**, *442*, 136158. https://doi.org/10.1016/j.cej.2022.136158.
